# Plasmon-Induced Transparency by Hybridizing Concentric-Twisted Double Split Ring Resonators

**DOI:** 10.1038/srep15735

**Published:** 2015-10-28

**Authors:** Mohammad Parvinnezhad Hokmabadi, Elizabath Philip, Elmer Rivera, Patrick Kung, Seongsin M. Kim

**Affiliations:** 1 Department of Electrical and Computer Engineering, The University of Alabama, Tuscaloosa, Alabama 35487, USA

## Abstract

As a classical analogue of electromagnetically induced transparency, plasmon induced transparency (PIT) has attracted great attention by mitigating otherwise cumbersome experimental implementation constraints. Here, through theoretical design, simulation and experimental validation, we present a novel approach to achieve and control PIT by hybridizing two double split ring resonators (DSRRs) on flexible polyimide substrates. In the design, the large rings in the DSRRs are stationary and mirror images of each other, while the small SRRs rotate about their center axes. Counter-directional rotation (twisting) of the small SRRs is shown to lead to resonance shifts, while co-directional rotation results in splitting of the lower frequency resonance and emergence of a PIT window. We develop an equivalent circuit model and introduce a mutual inductance parameter M whose sign is shown to characterize the existence or absence of PIT response from the structure. This model attempts to provide a quantitative measure of the physical mechanisms underlying the observed PIT phenomenon. As such, our findings can support the design of several applications such as optical buffers, delay lines, and ultra-sensitive sensors.

Metamaterials are well-known for their unusual optical responses such as negative refractive index, super-resolution imaging, and highly asymmetric and non-reciprocal behaviors that are impossible to elicit from naturally-occurring materials^1^,^2^,^3^,^4^. In addition to these unique characteristics, they can also emulate some of the renowned atomic and condensed matter phenomena such as electromagnetically induced transparency (EIT), Fano resonances, and orbital hybridization^5^,^6^,^7^. The former is indeed of significant importance owing to its exclusive feature of slow light which promises various applications in developing ultra-accurate sensors, low-power optical switches, optical buffers, and delay lines^8^,^9^,^10^,^11^,^12^,^13^,^14^. EIT is essentially a quantum destructive interference phenomenon which appears in a three level atomic system where a high-power pump beam induces a narrowband “dark” state to open a transparency window inside the broader absorption band of a “bright” state excited by a probe beam^15^,^16^. EIT has been observed in various media including cold atoms, warm atoms, and plasmas; however, cumbersome experimental conditions have often hampered its practical implementation^17^,^18^,^19^,^20^,^21^,^22^. The imitation of EIT by using metamaterials becomes significant where metamaterials can tremendously relax those experimental constraints. Since the first demonstration of EIT in metamaterials, called plasmon induced transparency (PIT), several metamaterial structures have been developed to achieve broadband, multiband, actively controllable, and polarization insensitive PIT from visible to microwave parts of the spectrum^7^,^23^,^24^,^25^,^26^,^27^,^28^,^29^,^30^,^31^,^32^,^33^. Most of those PIT structures leverage meta-atoms (i.e. building blocks of metamaterials) such as cut wires and/or split ring resonators (SRRs) as the analogues of bright or dark states when they either directly couple to the incident radiation (bright state) or are excited via induction from the bright meta-atom (dark state).

On the other hand, the interaction or hybridization of meta-atoms, when placed in a close proximity of one another^34^,^35^,^36^,^37^,^38^, tends to lead to the emergence of new resonant states from the splitting of degenerate modes. This can be easily achieved using SRRs and double split ring resonators (DSRRs) under various geometrical configurations in which coupling of both magnetic and electric moments can occur^39^,^40^,^41^,^42^,^43^,^44^,^45^,^46^,^47^,^48^,^49^. However, there are few reports of PIT through hybridization of SRRs, and the physical mechanisms underlying the interaction and emergence of PIT has not been studied previously.

In this work, we report the theoretical design and simulation, as well as experimental validation of a novel approach to specifically achieve and manipulate PIT by hybridizing two concentric-twisted DSRRs on flexible polyimide substrates. We further propose an equivalent circuit model to gain deeper understanding of the physics underlying the hybridization interaction, and therefore the occurrence or absence of PIT through hybridization, which we find to be quantitatively characterized by a mutual inductance parameter M and more specifically its sign. Finally, we experimentally determine a group delay of about 7 ps and relative group velocity of 0.06 associated with the proposed PIT structures, in good agreement with simulation results.

## Results

**Figure 1:**
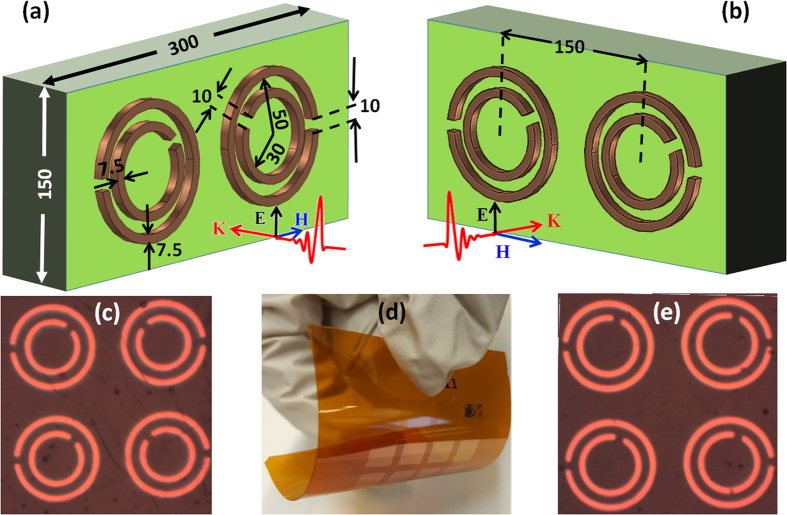
Schematic and optical microscope image illustrations of hybrid concentric twisted DSRRs. Schematic illustrations of counter-directional (**a**) and co-directional (**b**) structures. Optical microscope images of a fabricated sample of counter-directional (**c**) and co-directional (**e**) twisted DSRRs. An image of samples fabricated on a flexible 125 μm Kapton polyimide film (**d**).

Figure 1a,b represent schematic illustrations of the DSRR array unit cells studied in this work, which consist of hybridized counter-directional and co-directional twisted DSRRs, respectively (all dimensions in micrometer). Magnified optical microscope images of the correspondingly fabricated structures can be seen in Fig. 1c,e. The arrays were realized on flexible polyimide films as shown in Fig. 1d. As illustrated in the figures, the outer or large SRRs are fixed and mirror images of each other. In counter-directional structures (Fig. 1a) the inner or small SRRs rotate synchronously (symmetrically) to each other in opposite directions, while in co-directional structures (Fig. 1b) the small SRRs rotate synchronously in the same direction. We employed standard photolithography to realize 200 nm thick Cu SRR arrays deposited on a flexible 125 μm thick Kapton polyimide film substrate. The fabricated samples were characterized using terahertz time domain spectroscopic (THz-TDS) method in transmission mode. The THz radiation illuminates the arrays at normal incidence with the wave polarization oriented along the gap of the large SRRs in order to directly excite them as bright resonators at their fundamental frequency (see methods). The design and simulation of the spectral response of the structures was carried out using finite element numerical method (FEM), with periodic conditions (PBC) for all side boundaries perpendicular to the plane of the SRRs and perfectly matched layers (PML) for the front and back faces parallel to the plane of the SRRs. In the simulations, we used a conductivity of 6×10^7^ (S/m) for the Cu of the SRRs and a permittivity of 3.15 for the polyimide. For transmission measurements, we divided the transmission through the samples by that of a bare 125 μm Kapton polyimide substrate, which was used as a reference. For phase measurements and determination of the group delay and relative group velocity, we performed an air scan as the reference (see methods).

**Figure 2:**
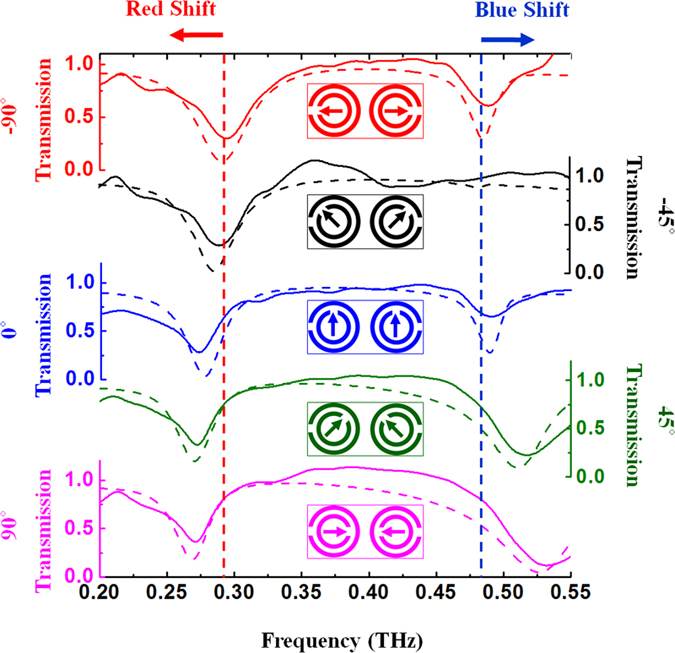
Transmission spectra of counter-directional DSRRs. Insets show the front view of the couther-directional twisted DSRRs. By rotating from −90° to 90°, the first resonance (lower frequency) bears a red-shift and the second resonance (higher frequency) experiences a blue-shift.

Figure 2 shows both measured (solid lines) and simulated (dashed lines) transmission spectra of counter-directional twisted DSRRs when the small SRRs in each DSRR are progressively rotated from −90˚ to 90˚ in opposite directions such that the gap in the small SRRs moves continuously far away from the gap in the large SRRs. An illustration of the unit cell is shown in the inset of its associated spectrum. As shown in Fig. 2, these counter-directional structures exhibit two distinct resonances: one at a lower frequency which stems from large SRRs and one at higher resonance frequency which primarily arises from the small SRRs. However, these resonances are not independent of each other: because of the interaction between the small and large SRRs within each DSRR, any physical change to one influences the resonance response of the other. In the present case, a small perturbation is achieved when rotating the small SRRs equally but in opposite directions as shown in Fig. 2, while the large SRRs remain stationary, in such a manner that the two DSRRs remain mirrors of each other at any rotation angle. When rotating the small SRRs from −90˚ to 90˚, the lower resonance (f_res.1_) is red shifted while the higher resonance (f_res.2_) experiences a blue shift. The resonance strength at the lower frequency remains fairly unchanged, but that at the higher frequency reduces gradually by rotation in the course of rotation from −90˚ to 90˚, and in the special case of −45˚ case the higher resonance completely disappears.

Furthermore, additional simulations have confirmed that using only one such DSRR (either one) instead of two in a unit cell does not change the resulting spectra in Fig. 2 (see supplementary material), which is essentially equivalent to changing one of the DSRRs in the unit cell with its mirror image with respect to a vertical plane since that would mean both DSRRs are the same.

The red and blue shifts in the resonance near f_Res.1_ of these counter-directional twisted DSRRs, with a fairly constant strength, are most interesting because they can be used to easily achieve PIT phenomenon in the following manner. Let us start by considering the 0˚ case as a reference structure (blue plot in Fig. 2), a rotation of the small SRRs in one direction (e.g. negative or toward −45˚ shown in Fig. 2) or the opposite direction (e.g. positive or toward +45˚ in Fig. 2) will cause the resonance near f_Res.1_ to shift to a slightly higher or lower frequency than the reference resonance, respectively. Consequently, a hybrid structure that contains both a “positive” and “negative” twisted DSRRs in the unit cell would exhibit two resonances slightly shifted from each other, which is essentially equivalent to a splitting of the original resonance and thus the potential emergence of a PIT transmission window. More precisely, since we saw previously that the spectral responses in Fig. 2 are unchanged when using only one of the 2 counter-directional twisted DSRRs, the desired hybrid structure exhibiting PIT can be realized with only two DSRRs instead of four: a negatively twisted DSRR such as the right one in the −45˚ inset in Fig. 2 (black), and a positively twisted DSRR such as the left one in the +45° inset in Fig. 2 (green). In other words, this configuration corresponds exactly to the case of co-directional twisting of small SRRs in the unit cell, as illustrated in Fig. 1b,e. This means that the co-directional twisting of the small SRR in these DSRR arrays can lead to a PIT window as a result of opposite shifts in the transmission resonance whereas the counter-directional twisting of the small SRRs only leads to a frequency shift in a single direction.

**Figure 3:**
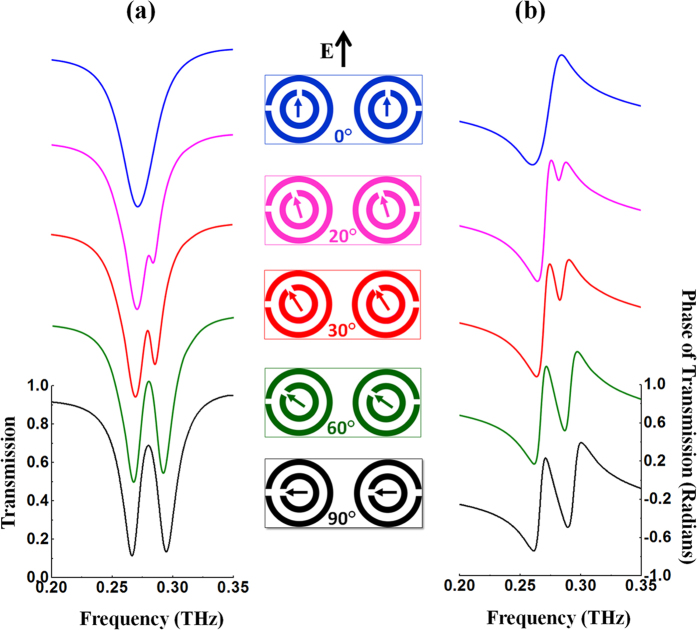
Simulated transmission and phase spectra of hybridized co-directional twisted DSRRs. Simulated transmission (**a**) and phase (**b**) spectra of hybrid co-directional twisted DSRRs when the small SRRs rotate synchronously in the same direction around their axes to achieve PIT. The middle column shows front view illustrations of the corresponding structures.

**Figure 4:**
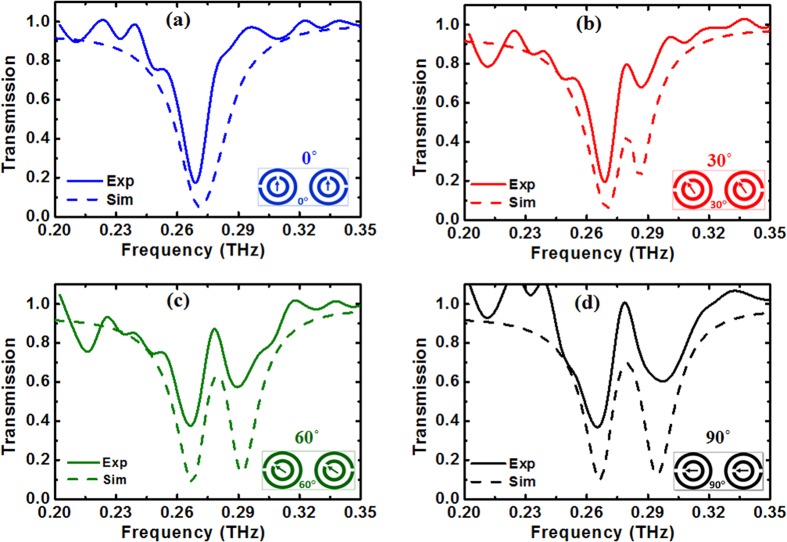
Comparison between simulated and experimental transmission spectra for co-directional twisted DSRRs. (**a**–**d**) transmission spectra for 0°, 30°, 60°, and 90° rotations of the small SRRs, respectively.

The effect of the rotation of the small SRRs on the opening of the PIT window in these co-directional twisted DSRRs is illustrated in Fig. 3 which shows the simulated transmission (Fig. 3a) and phase (Fig. 3b) spectra for rotation angles from 0° to 90°. At 90°, a PIT window can be clearly observed at ~0.280 THz. A comparison of the experimentally measured and simulated transmission spectra is depicted in Fig. 4 and a fairly good agreement is achieved.

**Figure 5:**
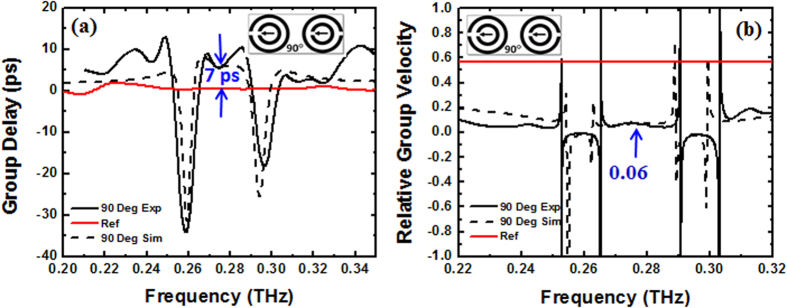
Comparison of simulated and experimental group delay and relative group velocity. (**a**) Group delay and (**b**) relative group velocity (V_g_/c) associated with 90° co-directional twisted DSRRs that exhibit PIT at ~0.280 THz. Solid and dashed black lines represent experimentally measured and simulated results, respectively. The red lines are group delay and relative group velocity of the reference polyimide.

The group delay and relative group velocity associated with the 90° co-directional twisted DSRRs that exhibit PIT are represented in Fig. 5. The solid and dashed black lines correspond to the values from experimentally measured and simulated transmission spectra, respectively. The solid red lines correspond to the group delay and relative group velocity of the polyimide reference. The group delay τ_g_ introduced by the metamaterial structure was calculated using


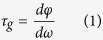


where φ and ω are the phase and angular frequency, respectively. To determine the phase φ of the transmission from the simulation spectra, we subtracted the phase of the incident wave, traveling in the air between the input port and surface of the structure, from the phase between the input and output ports. In doing so, only the desired phase difference between the front surface of the structure and the output port which is positioned 125 μm behind the DSRRs is obtained (see methods). However from the experimentally measured spectra, the phase of air scan (as the reference) was first subtracted from the measured phase of the sample, therefore an additional phase delay of air with a thickness of 125 μm (corresponding approximately to the thickness of the sample) was manually added to the subtraction (see methods). The relative group velocity V_g_ of the wave in the structure was subsequently obtained using


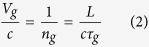


where c, n_g_, and L are the speed of light in vacuum, group index, and thickness of the metamaterial structure considered to be 125 μm.

From Fig. 5 we observe that at frequencies away from the resonances, the group delay and relative group velocity of THz radiation tends to be equal to that of polyimide, as expected. At the resonance frequencies of 0.265 THz and 0.295 THz the metamaterial demonstrates negative group delay and velocity. At around 0.280 THz where there exists a ~70% PIT transmission, the THz radiation experiences a delay of around 7 ps, while the group velocity of the THz wave reduces to nearly 0.06 times the speed of light in the vacuum.

## Discussion

**Figure 6:**
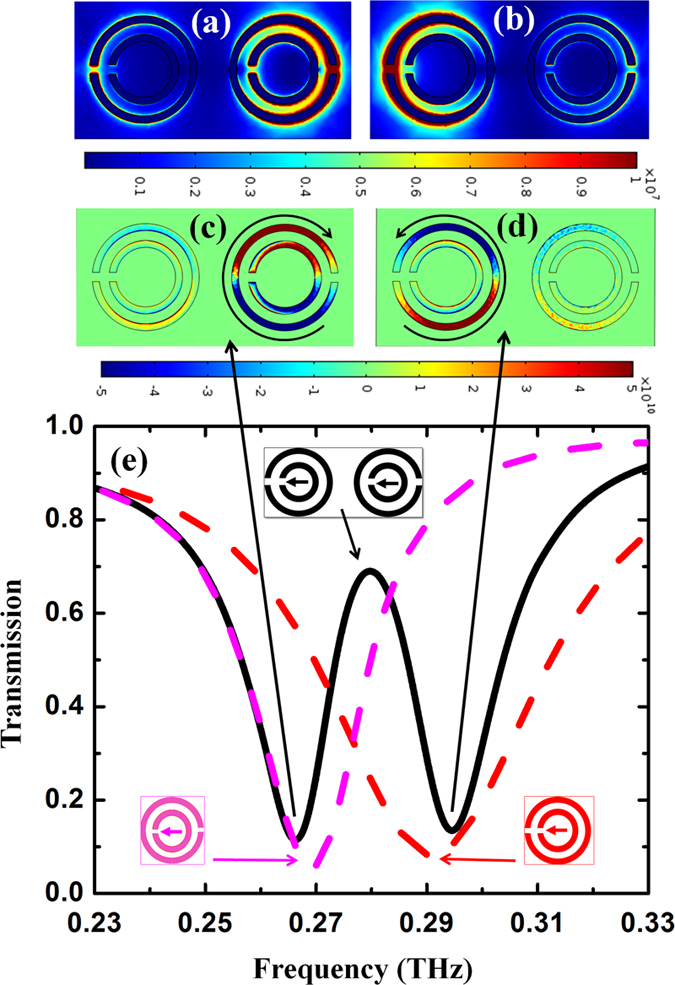
Comparison of transmission spectra between 90° co-directional PIT structure and its constituent DSRRs, electric field and current density distributions at transmission resonance frequencies. (**a**) Comparison of transmission spectra between 90°-co-directional PIT structure and its constituent elements. (**b**,**c**) Electric field strength distribution within the 90°-co-directional PIT structure at the two transmission resonance frequencies at 0.265 and 0.295 THz. (**d**,**e**) Current density distribution in the 90°-co-directional PIT structure at the same two resonance frequencies.

The observation of PIT in the 90° co-directional twisted DSRR structure of Fig. 4d can be interpreted by further examining the resonances of each constituent DSRR as well as their interactions, with the aid of our FEM analysis. Figure 6e compares the transmission spectrum of the PIT structure (solid black line) superimposed with that of its two constituent DSRRs (pink and red lines, respectively). The electric field strength distribution in the structure is shown in Fig. 6a,b at the two resonance frequencies of 0.265 THz and 0.295 THz, respectively, along with their associated current densities in Fig. 6c,d. It is clearly observable that the two minima in the transmission spectrum of the PIT structure stem from the resonances associated with each of the constituent DSRRs. Although they are relatively broad at 0.270 and 0.290 THz, both resonances become narrower and a transparency window appears at 0.280 THz due to interaction between the two DSRRs after hybridization. Interestingly as a result, unlike conventional EIT phenomena where bright and dark states with a large difference in resonance bandwidths are required, the PIT demonstrated here is eventually created by coupling two bright states with comparable bandwidths.

**Figure 7:**
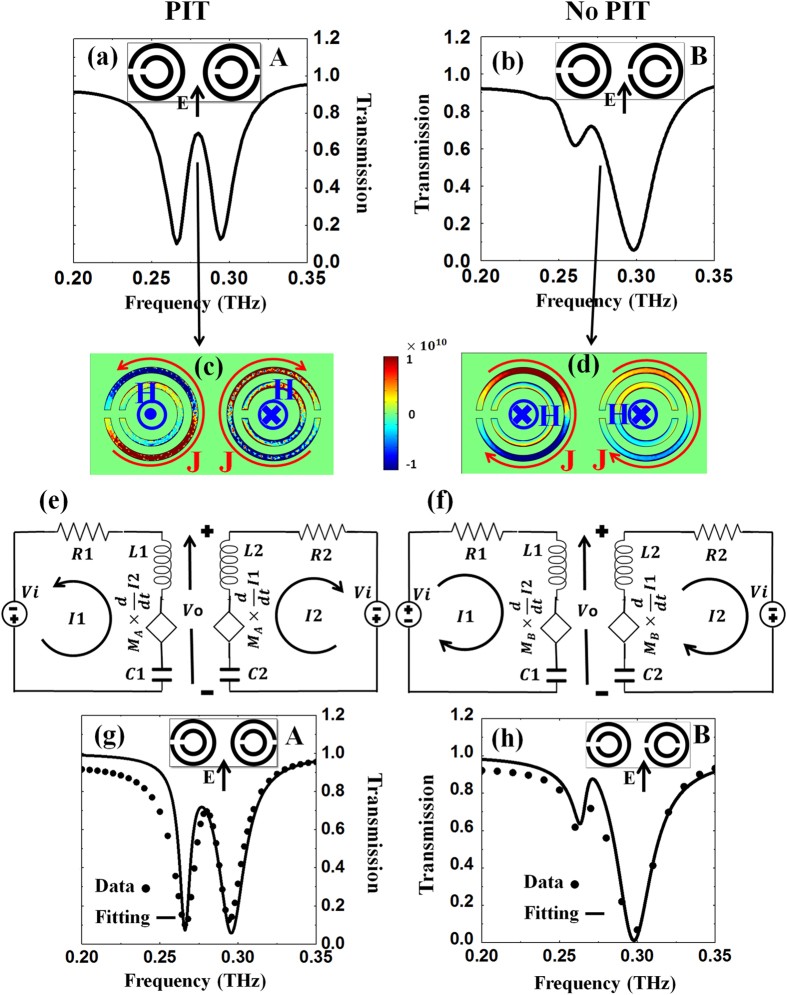
Transmission spectra, electric current at 0.280 THz, circuit model, and fitted spectra of structures A and B. (**a**,**b**) are simulated transmission spectra of structure A and B. (**c**,**d**) are their associated current densities (x component) where red vectors, labeled J, and blue centrifugal and centripetal marks, labeled H, are used to show current and magnetic field direction of large SRRs. (**e**,**f**) Electric circuit models of structure A and B. (**g**,**h**) fitted transmission spectrum for structure A and B respectively where dots show the simulated data and solid lines are fitting result by the model.

By contrast to the hybridized counter-directional DSRRs, in the co-directional DSRRs interaction between the two constituent DSRRs is essential to the emergence of a PIT response. For example, if one of the DSRRs in the PIT structure is rotated by 180˚ about its axis (e.g. right DSRR), the two adjacent DSRRs will interact with each other in such a manner that the higher frequency resonance will dominate over the lower frequency one, leading to disappearance of the PIT transmission window as shown in the spectrum comparison in Fig. 7a,b.

For the rest of manuscript, we will call “structure A” the 90˚ co-directional PIT structure in Fig. 7a and “structure B” the other one in Fig. 7b. The current densities (x component) of structures A and B at 0.280 THz are illustrated in Fig. 7c,d, respectively. For clarity, we used red arrows and a label J, to show the current directions in the large SRRs, and blue into-plane (×) and out-of-plane (.) symbols for the induced magnetic fields. We clearly see that the electric currents generated in the two large SRRs of structure A are in opposite direction while they are in the same direction for structure B. Correspondingly, the generated magnetic fields inside the DSRRs are in opposite directions for structure A and in the same direction for structure B.

To gain a better understanding of the physics underlying these observations, we propose the equivalent circuit model for structures A and B, shown in Fig. 7e,f respectively. Each DSRR is modeled as a series RLC circuit with R as the resistance, C its capacitance, and L its self inductance. The resonance frequencies 
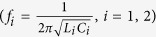
 of the left and right RLC circuits are set at 0.270 THz and 0.290 THz, which correspond to the resonances observed in Fig. 6 (red and pink lines). The coupling of an incident THz wave into the DSRRs is represented by a voltage source, V_i_, with the same magnitude and phase in each circuit since the wave is arriving at normal incidence. To represent the mutual interaction between the two DSRRs, we introduce a the mutual inductance M such that an additional dependent voltage source equal to 
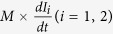
 is added in the circuit, i.e. the voltage in one loop being proportional to the rate of change in the current inside the other loop. Since the DSRRs of structure A are exactly the same as structure B when considered individually, their circuit RLC models will be identical in both structures. However, due to the currents (or magnetic fields) being antiparallel in structure A and parallel in structure B (Fig. 7c,d), the interaction between the DSRRs in each unit cell will not be similar for the two structures and we expect the coupling parameter M to have different values (M_A_, M_B_) to characterize the dissimilar responses of A and B.

The equations governing this circuit models can be summarized as


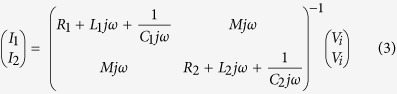


where M is either M_A_ for structure A or M_B_ for structure B, and ω is the angular frequency. Equa-tion (3) is used to extract the transfer function (V_O_/ V_i_) of the circuit versus frequency. Fitting the square magnitude of transfer function to the simulated transmission spectra of structures A and B is shown in Fig. 7g,h respectively, and results in the values of the circuit elements and coupling parameters summarized in Table 1.

As anticipated, all circuit element parameters (R, L and C) have almost identical values for structures A and B. A remarkable difference is the value of M, which is found to be positive for structure A while it is negative for structure B but with nearly the same magnitude.

The near-equality of all parameters other than M confirms that the PIT and not-PIT responses of structures A and B originates solely from different coupling schemes of their constituent DSRRs. The opposite signs of M resembles an analogous behavior between two solenoids: like two solenoids with magnetic fluxes in opposite directions, structure A gives rise to a positive (attractive) coupling factor whereas, similar to two solenoids with magnetic fluxes in the same direction, structure B gives rise to a negative (repulsive) coupling factor. In other words, the mutual (due to M) and self induced (due to L) currents of each large SRR are in the same direction for structure A but in opposite directions for structure B. Thus, a positive mutual inductance M_A_ between DSRRs in structure A will increase the total inductance (L_i_+M_A_) in each DSRR. As a result, since the bandwidth of the resonance in a series RLC circuit is equal to R/L, the bandwidth of the resonances from the DSRRs is expected to be reduced when coupled, which is consistent with what was observed in Fig. 6: both resonances became narrower, which gave rise to the opening of the PIT transmission window in between, at 0.280 THz. By contrast, a negative mutual inductance M_B_ in structure B leads to the opposite effect: a reduction of the total inductance of both DSRRs, a broadening of their transmission resonance, and since the higher frequency resonance becomes dominant over the other one, the PIT window diminishes in structure B.

## Methods

### Numerical Simulation

**Figure 8:**
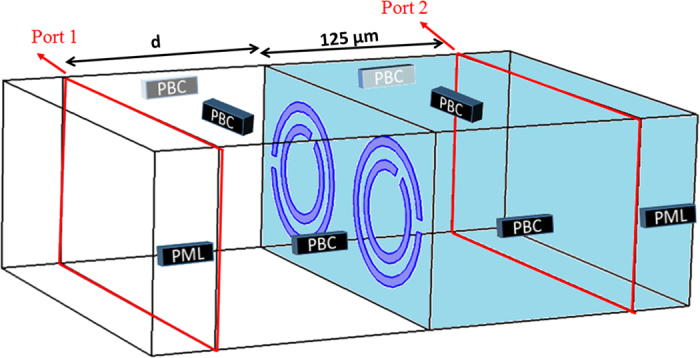
Schematic illustration of simulation environment and conditions. Blue area is polyimide and the white area contains air.

In the design of the structures, we used Comsol Multiphysics FEM method to solve Maxwell equations. Figure 8 shows a schematic illustration of the structure under simulation with the details highlighted. We utilized PBC for all side boundaries perpendicular to the plane of the SRRs, and PML was applied for front and back boundaries which are parallel to the plane of the SRRs. The incident wave was launched at normal incidence with its polarization along the gap of large SRRs such that first order resonance was excited in the SRRs in all structures. Input and output ports were implemented to measure phase and transmission spectra by utilizing scattering parameters. The transmission T was calculated by using the scattering parameter S_21_ through:


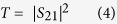


To calculate the phase of transmission, we subtracted the phase of the incident wave inside the air between the input port and the surface of the structure from the phase between the two ports, so that only the desired phase difference between the front surface of the structure and the output port which is positioned 125 μm behind the SRRs is obtained:


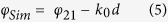


where φ_Sim_ is the phase of transmission through the sample, φ_21_ is the phase difference between input and output ports, k_0_ is the wavenumber in vacuum, and d is the distance between port 1 (input port) and surface of the sample. The group delay was then calculated using Eq. (1) and subsequently the relative group velocity was evaluated using Eq. (2) where 125 μm was considered for the thickness of metamaterial (L).

### Measurement

**Figure 9:**
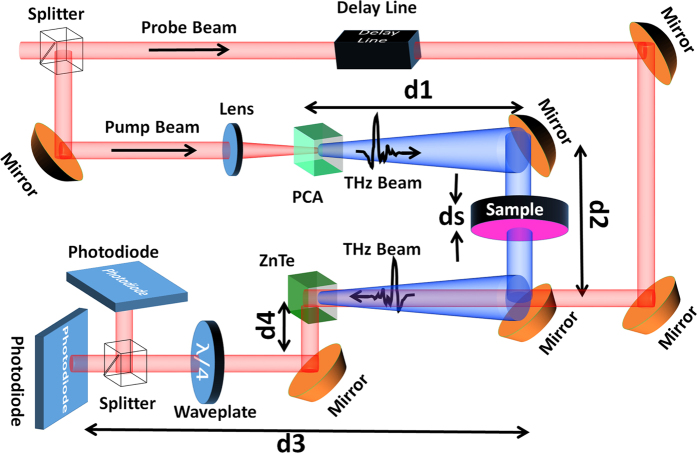
Schematic representation of THz-TDS setup used to characterize samples. A photoconductive antenna generates THz radiation and an electro-optic scheme is used for detecting the transmitted THz radiation through the sample.

For experimental measurements, a THz-TDS system in transmission mode was used. Figure 9 shows a schematic illustration of the system. A 780 nm excitation laser beam with a repetition rate of 76 MHz is split into pump and probe beams. The pump beam excites a photoconductive antenna (PCA) to generate linearly polarized THz wave. For detection, an electro-optical sampling method is applied such that both transmitted THz wave through the sample and the 780 nm probe beam meet each other at a *birefringent* ZnTe crystal by controlling the timing between them through a delay stage. The THz wave induces *different refractive indices* along two orthogonal optical axes in the crystal, which affects the polarization of the time-delayed probe beam that passes through it. Then, a splitter followed by a waveplate separates two orthogonal polarizations of the probe beam and subsequently two photodiodes are used to detect each split beam. The detected voltage difference by photodiodes is then amplified by a lock-in amplifier which is a representation of the magnitude and phase of the transmitted THz wave through the sample.

The transmission ratio was obtained by dividing the transmitted THz wave through the samples by that obtained through a piece of the same (but bare) Kapton polyimide substrate film used as the reference. However, to evaluate the phase change between the exterior surfaces of the samples (φ_Sample_), the phase difference between the incident THz wave and the detected THz wave transmitted through sample (φ_measure_) was first measured. Then, the phase of air and associated optical elements such as lenses or mirrors (φ_mirrors_) had to be subtracted from φ_measure_ according to:


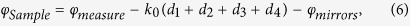


where k_0_ is the wave number of air and d_1_ + d_2_ + d_3_ + d_4_ is the distance between the photodiodes and the PCA (Fig. 9). Therefore, an air scan was performed as the reference where the corresponding phase change φ_AirScan_ includes 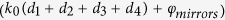. This phase was then subtracted from the measured phase of sample (φ_measure_). However, while doing so, we also subtract a phase change associated with the contribution of air with the same thickness as the sample (d_s_). Therefore, a phase change equal to k_0_d_s_ needs to be added back, which leads to a total phase change through the metamaterial sample to be:


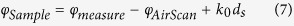


## Additional Information

**How to cite this article**: Parvinnezhad Hokmabadi, M. *et al.* Plasmon-Induced Transparency by Hybridizing Concentric-Twisted Double Split Ring Resonators. *Sci. Rep.* **5**, 15735; doi: 10.1038/srep15735 (2015).

## Supplementary Material

Supplementary Information

## Figures and Tables

**Table 1:** Evaluated elements of circuit model resulted from fitting simulated transmission spectrum.

Structure	C_1_ [fF]	C_2_ [fF]	L_1_ [pH]	L_2_ [pH]	M [pH]	R_1_ [Ω]	R_2_ [Ω]
A	3.53	22.80	98.44	13.21	3.10	4.70	1.90
B	3.55	22.87	98.61	13.17	−3.20	4.50	2.20
