# Supplementary material for: Plasmon-Induced Transparency by Hybridizing Concentric-Twisted Double Split Ring Resonators
Source: Sci Rep. 2015 Oct 28;5:15735. doi: 10.1038/srep15735 (PMC4623747; doi:10.1038/srep15735)
Supplement: Supplementary Information [file srep15735-s1.doc]

**Plasmon-Induced Transparency by Hybridizing Concentric-Twisted Double Split Ring Resonators**

**Mohammad Parvinnezhad Hokmabadi^1^, Elizabath Philip^1^, Elmer Rivera^1^, Patrick Kung^1^, and Seongsin M. Kim^1*^**

^1^Department of Electrical and Computer Engineering, The University of Alabama, Tuscaloosa, Alabama 35487, USA

*Correspondence and requests for materials should be addressed to S.M.K. (email: [seongsin@eng.ua.edu](mailto:seongsin@eng.ua.edu)).

**Coupling between DSRRs in counter-directional structure**

Here, we illustrate the simulations results when the center to center distance between DSRRs in counter-directional twisted structures is 120 µm (two DSRRs are very in the unit cell close to each other) and when there exists only one DSRR in the unit cell. Figures 1S (a) and 1S (b) demonstrate the simulated transmission spectrum for these two structures, respectively. As it is observed in the figures, all characteristics associated with counter-directional structures such as the red and blue shift of resonance frequencies and disappearing of the second resonance at -45˚are preserved, which verifies that the aforementioned characteristics are as a result of interaction between small and large SRRs in the unit cell and the interaction between DSRRs does not affect these characteristics.


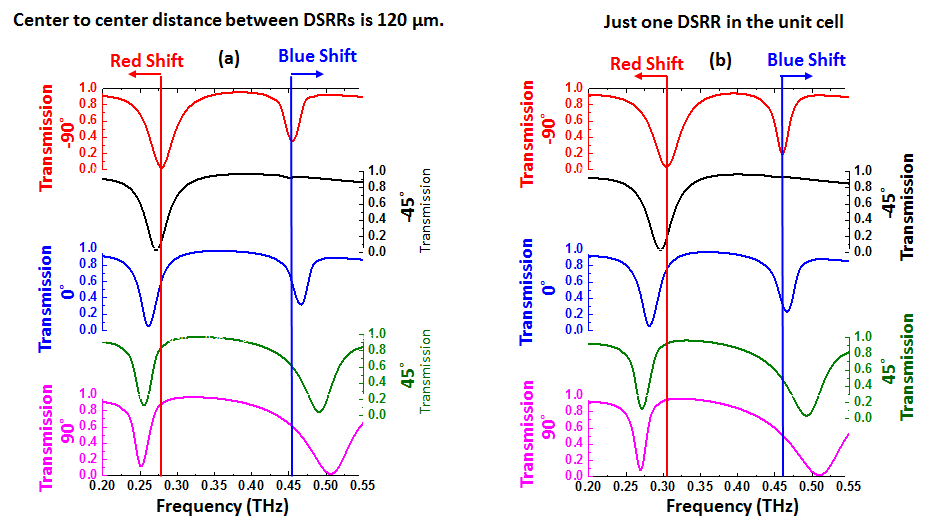


**Figure 1S. Transmission spectrum of counter directional structures demonstrating the effect of coupling between DSRRs.** (a) when center to center distance between DSRRs is 120 µm and (b) when there is just one DSRR in the unit cell.

**Hybridized counter-directional twisted DSRRs**

To enlighten the origin of the red and blue shifts of f_res.1_ and f_res.2_, we analyze the current density distributions at resonance frequencies in SRRs for two cases of -90° and 90° rotations which are obtained by simulation. Figures 2S (a) and 6e show the x component of current densities in SRRs for -90˚ structure at f_res.1_ and f_res.2_ frequencies, respectively, and Figs. 2s (c) and 2S (g) illustrate corresponding current densities at f_res.1_ and f_res.2_ for 90˚ case. A schematic representation of SRRs have been demonstrated in Figs. 2S (b), 2S (d), 2S (f), and 2S (h) to elucidate the direction of created currents in SRRs (black circular vectors) and their associated magnetic fields (green centrifugal (×) and centripetal (.) signs). The created resonances in all structures are the first order resonances in which an electric dipole moment is formed within the gap with opposite poles on either side of it (blue - and + signs in figures). The first order resonating currents in SRRs can be found by direct coupling of the incident electromagnetic wave into SRRs if the incident electric field has a component along the gaps, or through the induction from another SRRError: Reference source not found^, 49^. f_Res.1_ in our structure is an example of former case which is mainly due to the coupling of the incident electric field into the gap of large SRRs although their resonance frequencies are altered by rotating the small SRRs. The latter case is found in the structure with 0° rotation where the gap of small SRRs is orthogonal to the incident electric field. In this case, the observed resonance at f_Res.2_ is merely due to the induction from large SRRs. It is worth mentioning that current density in small SRRs shown in Fig. 2S increases continuously when they are rotated from -90° toward 90°. A similar behavior is seen at f_Res.2_ for large SRRs. Nonetheless, the major difference between f_Res.1_ and f_Res.2_ is the direction of currents in small and large SRRs. At f_Res.1_, the currents in small and large SRRs are in the same direction, while at f_Res.2_, they are in opposite directions. This is in fact the primary reason for red shift of f_Res.1_ and blue shift of f_Res.2_ in the course of rotation from -90˚ to 90˚, by considering the magnetic fields created by those currents. Figure 2S (i) displays the energy level scheme to interpret the shift of resonance frequencies depending on the type of interaction between electric, or magnetic moments. According to the energy level diagram, parallel moments will repel each other leading to an increase in the restoring force between them and hence frequency blue shift, while anti-parallel moments will attract each other resulting in a decrease in the restoring force between them and thus frequency red shiftError: Reference source not found^, 49^.


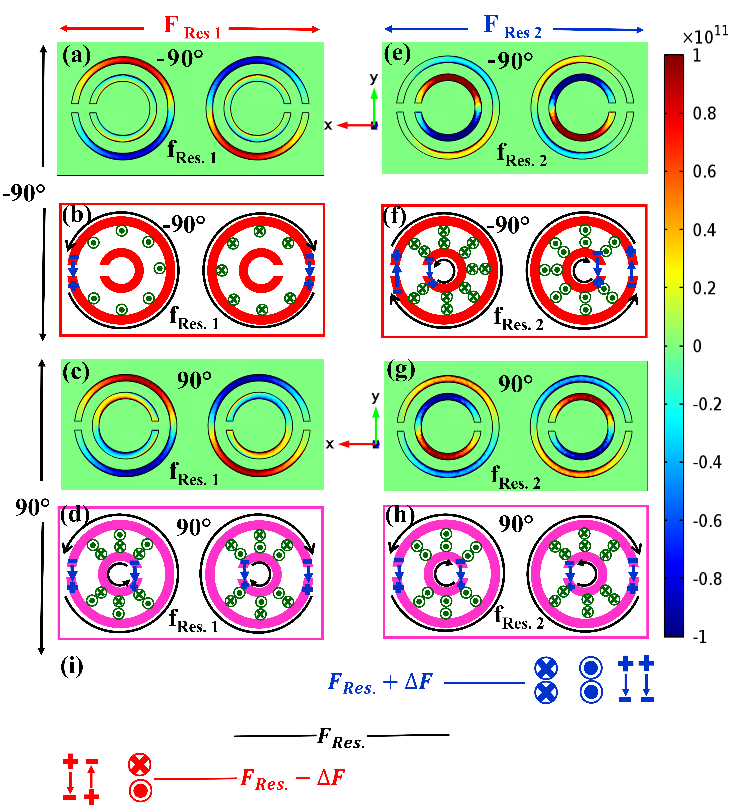


**Figure 2S. Current density distribution and the interaction between created magnetic an electric moments.** Current density of SRRs at the first resonance in -90° structure (a) and in 90° structure (c). Schematic representation of SRRs demonstrating current density (black arrow) and the created magnetic and electric moments at first resonance in -90° structure (b) and in 90° structure (d). Current density of SRRs at the second resonance in -90° structure (e) and in 90° structure (g). Schematic representation of SRRs demonstrating current density (black arrow) and the created magnetic and electric moments at the second resonance in -90° structure (f) and in 90° structure (h). i. The energy level diagram to interpret shift of resonance frequencies according to the interaction between moments.

From Fig. 2S (a), it is observable that the current in small SRR in -90˚ structure is negligible at f_Res.1_. Therefore, there exist no or the least interaction between the magnetic (electric) moments of small and large SRRs (Fig. 2S (b)). However, at the same resonance frequency, f_Res.1_, by moving toward 90˚ structure (Fig. 2S (c)), the current density in small SRRs continuously increases and the created magnetic (electric) moments between large and small SRRs (along the gaps) oscillate in opposite directions. Consequently, according to the energy level scheme the resonance frequency will relatively red shift depending on the strength of magnetic moments between SRRs and the direction of interacting electric moments relative to each other. Like the first resonance (f_Res.1_), the created currents at f_Res.2_ in large SRRs and hence their associated magnetic field increase by rotating small SRRs from -90˚ to 90˚ (Figs. 2S (e) and 2S (g)), but in contrast to f_Res.1_, the magnetic moments between SRRs are oriented in the same direction (Figs. 2S (f) and 2S (h)). It is also noticeable that the electric moments of small and large SRRs at -90˚ case are oriented in opposite directions (Fig. 2S (f)), whilst at 90˚structure they possess the same direction (Fig. 2S (h)). Thus, conforming energy level diagram, f_Res.2_ will experience a blue shift and again the amount of this blue shift depends on the strength of the magnetic moments between SRRs and the orientation of electric moments of SRRs interacting with each other.


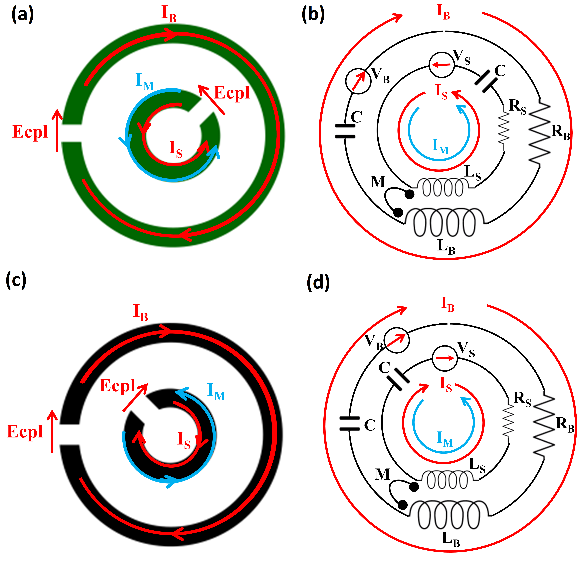


**Figure. 3S. Schematic illustrations of half of the unit cells for 45° and -45° counter directional twisted DSRRs and their associated electric models**. Schematic illustration of 45° (a) and -45° (c) structure with their coupled (I_B_) and induced (I_S_) currents and their associated electric model respectively in (b) and (d).

The absence of the second resonance at -45˚ structure and the reduction of transmission at this resonance by twisting the small SRR toward 90˚ can be interpreted by means of an electric model. For the sake of this interpretation, we focus in two cases of rotation of small SRRs by 45˚ and -45˚. The others can be interpreted in a similar manner. Figure 3S (a) represents the half of the unit cell of the 45˚ rotated structure with its equal circuit model displayed in Fig. 3S (b). Correspondingly, the half of the unit cell of -45˚ rotated structure and its equal circuit model have been demonstrated in Figs. 3S (c) and 3S (d). We model each SRR as a RLC circuit, where R_S_ and R_B_ are respectively the resistances of the small and large SRRs, and L_S_ and L_B_ are their corresponding inductances. Since both SRRs possess the same width, thickness, and gap dimensions, their equivalent capacitances will be equal which are denoted by C. While the incident electric field fully couples into the large SRR (E_cpl_) and creates current I_B_, it partially couples (√2/2E_cpl_) into the small SRR that excites the resonating current I_S_. To model this coupling scheme, two voltage sources are used to mimic the coupling of the incident electric field into small (V_S_) and large (V_B_) SRRs. Since large SRR is not rotating, V_B_ will maintain a constant value and direction, but the value and direction of V_S_ will change dependent on the rotation of small SRR. Ultimately, the interaction between two SRRs is modeled as a mutual inductance denoted by M. This mutual inductance will induce a current on each SRR in the opposite direction of its source current. For instance, the inducted current from the large SRR into the small one is represented by I_M_ which is in opposite direction of I_B_. It should be noted that a similar current will be inducted into the large SRR from the small one which has not been demonstrated in the figure. When the rotation angle of small SRR is between -90˚ and 0˚, the incident electric field couples into the gap of small SRR in such a manner that the excited current I_S_ is in the opposite direction of I_M_ as shown in the Figs. 3S (c) and 3S (d). In the special case of -45˚ structure, these two anti-parallel currents are of the same magnitude and therefore they cancel out each other leading to disappearance of the second resonance. Contrarily, when the rotation angle is between 0˚ and 90˚, both I_S_ and I_M_ possess the same orientation shown in the Figs. 3S (a) and 3S (b), therefore they will amplify each other giving rise to a gradual decrease in the transmission of the second resonance in the course of rotation of small SRR from 0˚ towards 90˚.
